# 

**Published:** 1886-08

**Authors:** 


					﻿'"Vol. VII.
AUGUST, 1886.
No. 8.
THZ
« wniFram nonti
A Monthly JFojjmwjli
DEVOTED TO
DENTAL AND ORAL SCIENCE.
W. C. BARRETT, M. D., D. D. S.,
EDITOR.
The Iew York Dental Journal Association,
PUBLISHERS,
ISTo. 35 West 46th Street, IXTew York.
AND
No. 208 Franklin St., Buffalo, N.Y.
$2.50 Per Annum in Advance, .... Single Copies, 25 Cents.
Entered at the Post-Office, Buffalo, N. Y., as Second-Class matter.
				

